# Anti–Peri-implantitis Bacteria's Ability of Robusta Green Coffee Bean ( *Coffea Canephora* ) Ethanol Extract: An *In Silico* and *In Vitro* Study

**DOI:** 10.1055/s-0042-1750803

**Published:** 2022-09-08

**Authors:** Alexander Patera Nugraha, I Gusti Aju Wahju Ardani, Ratri Maya Sitalaksmi, Nastiti Faradilla Ramadhani, Desi Rachmayanti, Dina Kumala, Viol Dhea Kharisma, Desintya Rahmadani, Martining Shoffa Puspitaningrum, Yuniar Rizqianti, Muhammad Dimas Aditya Ari, Albertus Putera Nugraha, Tengku Natasha Eleena binti Tengku Ahmad Noor, Muhammad Luthfi

**Affiliations:** 1 Dental Implant Group, Faculty of Dental Medicine, Universitas Airlangga, Surabaya, Indonesia; 2 Department of Orthodontics, Faculty of Dental Medicine, Universitas Airlangga, Surabaya, Indonesia; 3 Postgraduate Department of Dental Health Science, Faculty of Dental Medicine, Universitas Airlangga, Surabaya, Indonesia; 4 Department of Prosthodontics, Faculty of Dental Medicine, Universitas Airlangga, Surabaya, Indonesia; 5 Department of Dentomaxillofacial Radiology, Faculty of Dental Medicine, Universitas Airlangga, Surabaya, Indonesia; 6 Department of Biology, Faculty of Mathematic and Natural Science, Universitas Brawijaya, Malang, Indonesia; 7 Faculty of Dental Medicine, Universitas Airlangga, Surabaya, Indonesia; 8 Faculty of Medicine, Universitas Airlangga, Surabaya, Indonesia; 9 Membership of Faculty of Dental Surgery, Edinburgh University, United Kingdom; 10 Malaysian Armed Forces Dental Officer, 609 Armed Forces Dental Clinic, Kem Semenggo, Kuching, Sarawak, Malaysia.; 11 Oral Biology Department, Faculty of Dental Medicine, Universitas Airlangga, Surabaya, Indonesia

**Keywords:** dentistry, infectious disease, medicine, communicable disease, dental implant

## Abstract

**Objective** This study was aimed to investigate RGCBE extract as antioxidant and anti–peri-implantitis bacteria through *in vitro* study and its potential as antioxidant, antibacterial, anti-inflammatory, antibone resorption, and proosteogenic through *in silico* study.

**Materialsand Methods** Absorption, distribution, metabolism, excretion and toxicity prediction, molecular docking simulation, and visualization of chlorogenic acid (CGA) and coumaric acid (CA) as anti-inflammatory, antioxidant, and antibacterial were investigated *in silico* . Inhibition zone by diffusion method, minimum inhibitory concentration (MIC), and minimum bactericidal concentration (MBC) of RGCBE extract against *Aggregatibacter actinomycetemcomitans* (Aa), *Porphyromonas gingivalis* (Pg), *Fusobacterium nucleatum* (Fn), and *Prevotella intermedia* (Pi) were done.

**Statistical Analysis** the analysis of variance (ANOVA) difference test, and the post-hoc Tukey's Honest Significant Different (HSD) with a different significance value of *p* <0.05

**Results** GCA and CA compounds are good drug molecules and it has low toxicity. Chlorogenic acid have higher binding activity than coumaric acid to tumor necrosis factor (TNF)-α, nuclear factor (NF)-κB, receptor activation NF-κB (RANK) and its ligand (RANKL), interleukin (IL)-6, IL-10, runt related transcription factor (RUNX2), receptor activator nuclear Kappa beta Ligand-osteoprotegrin osteocalcin (RANKL-OPG), osteocalcin, nuclear factor associated T-cell 1 (NFATc1), tartate resistant acid phosphatase (TRAP), peptidoglycan, flagellin, dectin, Hsp70, and Hsp10 protein. RGCB ethanol extract has high antioxidant ability and it has MIC, MBC, and inhibit the growth of Aa, Pg, Fn, and Pi at 50% concentration with significantly different ( *p* =0.0001 and<0.05).

**Conclusion** RGCB ethanol extract has high antioxidant ability and 50% RGCB ethanol extract may act as strong anti–peri-implantitis bacteria *in vitro.* In addition, CGA in RGCB potential as antioxidant, antibacterial, anti-inflammatory, antibone resorption, and proosteogenic *in silico* .

## Introduction

Malocclusion is a sort of connection between the maxilla and mandible that differs from the usual form but is nonetheless considered normal. 1 Malocclusion is one of the most common oral health problems, second only to caries and periodontitis, according to the World Health Organization. In children and adolescents, prevalence ranges from 39 to 93%. This vast and varied prevalence range could be attributable to ethnic and age inequalities. 2 The prevalence of malocclusion in Indonesia is around 80% of the population and may increase dental and oral health problem based on basic health research or Riset Kesehatan Dasar in 2018. 3 Because of the physiological and social changes caused by this illness, the persistence of malocclusion with no therapy will result in significant consequences in the quality of life of children and their parents. 2 There could also be problems with esthetics, mastication, and phonation. About 46% young people with malocclusion had a negative impact on their oral health–related quality of life (OHRQoL) because they are more concerned about facial esthetic. 4 In Indonesia, there are only 0.7% malocclusion patient who receive orthodontic treatment. This has resulted in a high demand for orthodontic treatment services. 5

The convenience of mini-implants for orthodontists, as well as the broad acceptance of mini-implants by patients, has contributed to an increase in their popularity. In the realm of orthodontic mini-implants, there has been significant increase, mostly in design, in implantation techniques and a better understanding of the risk factors. Mini-implants are less bulky than prior anchorage methods, such as mini-plates, and can thus be implanted between the roots of teeth or in alveolar bone. Mini-implants have been proven to be successful in 80 to 100% of cases. Compared with palatal mini-implants, buccal mini-implants have a lower success rate. 6 Despite its promising success, effectiveness, and application, dentists rarely or never employ mini-implant devices. 7 Moderate production of proinflammatory cytokines is essential in physiological circumstances to maintain low-grade inflammation and proper mini-implant osseointegration. Excessive production of these mediators, on the other hand, may increase the risk of peri-implantitis and mini-implant failure by triggering a stronger inflammatory response, disrupting the balance of bone resorption required for mini-implant osseointegration, and ultimately raising the risk of peri-implantitis and mini-implant failure. 8

Bacterial infections are a common cause of endoosseous mini-implant failure. Periodontitis and peri-implantitis are both caused by the same bacterial species. Peri-implantitis is a polymicrobial illness that is mixed and changeable but is dominated by gram-negative anaerobic bacteria. 9 *Aggregatibacter actinomycetemcomitans* (Aa), *Porphyromonas gingivalis* (Pg), and *Prevotella intermedia* (Pi) were discovered to be more frequent in peri-implantitis than in peri-implant health. 10 Pg produces virulence factors like capsules, fimbriae, and proteases that aid colonization and the activation of dysbiotic inflammatory responses. On human periodontal epithelial cells, the capsule plays an important function in adhesion. Fimbriae have an adhesive role on oral surfaces in addition to their other functions. Gingipains, for example, are implicated in the colonization of the periodontal pocket. 11

Peri-implantitis has been shown to cause systemic changes at a variety of levels, including blood cell count, serum biochemical parameters, and cytokine levels, all of which can affect systemic conditions and disease. The inflammatory response in the peri-implant lesion is likewise more intense than the inflammatory response in the periodontal ulcer. 12 A new animal study discovered a link between peri-implant illness and changes in the total blood count. Chronic disease anemia (CDA) is thought to be mediated by cytokines that released as a result of the inflammatory process. 13

Antibiotic is being the recent therapy for peri-implantitis to against bacteria which cause peri-implantitis. Bone defect configuration, plaque control accessibility, and patient's medical condition may be the factors to consider for the treatment. Plaque control and host modulation are also important and helpful for controlling peri-implantitis management. Resective surgery is also needed for peri-implantitis treatment when pocket is found. Unfortunately, antibiotic usage for peri-implantitis may be not too effective because of antibiotic resistance for several patients, while resective surgery is an invasive procedure for patient if bone defect has occurred through the peri-implantitis. So, biomaterial exploration is needed for innovation of peri-implantitis antibacterial agent which also may be the host modulation. 14

Robusta green coffee bean (RGCB; *Coffea Canephora* ) is one of potential plants that may be used as herbal medicine for peri-implantitis antibacterial agents. Indonesia is an agricultural or a green economy country which has a variety of coffee beans, such as green beans. Indonesia's production of green coffee beans is one of the best quality in the Southeast Asia which has been the most green coffee bean exporter in Singapore. Jember RGCB contains many phenolic compounds which have beneficial effects as a biomaterial. Chlorogenic acid and coumaric acid are two of the most active compound in green coffee. *C. canephora* green coffee reported has chlorogenic acid level is more than *Coffea arabica.* CGA is the major polyphenol in 7 to 11% beside of caffeine in green coffee. 15 16 As the major polyphenol in green coffee, it has many pharmacological properties which is beneficial to be a biomaterial for herbal medicine. CGA contains in green coffee may has antioxidant activity. 17 Another compound in green coffee is coumaric acid which play a role to inhibit bacterial growth such as *Staphylococcus aureus* . 18 Coumaric acid also has antioxidant effect which may control anti-inflammatory cytokine through the inflammation responses. 19

However, the study about ethanol extract of RGCB ( *C. canephora* ) as antioxidant, anti-inflammatory, antibone resorption, proosteogenic, and antibacterial of peri-implantitis is still limited. RGCB ( *C. canephora* ) may promising phytotherapy that possessed antioxidant, anti-inflammatory, antibone resorption, proosteogenic and antibacterial ability that can treat peri-implantitis. Furthermore, in this study investigated RGCB ( *C. canephora* ) ethanol extract as antioxidant and anti–peri-implantitis bacteria through *in vitro* study and its potential as antioxidant, antibacterial, anti-inflammatory, antibone resorption, and proosteogenic through *in silico* study.

## Materials and Methods

### Ethical Clearance Statement

All methods in this research were performed in accordance with the relevant guidelines and regulations by Ethics Committee, Faculty of Dental Medicine, Universitas Airlangga, Surabaya, Indonesia, with number: 136/HRECC.FODM/III/2022.

### Bioinformatic Approach, an *In Silico* Study

#### Sample Preparation

This study used a chemical compound containing green coffee which consisted of chlorogenic acid and coumaric acid, information on the canonical three-dimensional (3D), and SMILE structures of the two compounds and was obtained from the PubChem database ( https://pubchem.ncbi.nlm.nih.gov/ ), then for protein. The targets used in this study were tumor necrosis factor (TNF)-α, nuclear factor (NF)-κB, receptor activation NF-κB (RANK) and its ligand (RANKL), interleukin (IL)-6, IL-10, RUNX2, RANKL-OPG, Osteocalcin, NFATC1, TRAP, peptidoglycan, flagellin, dectin, Hsp70 and Hsp10, 3D structure information, visualization method, Uniprot ID, protein data bank (PDB) ID, resolution (Å), weight (kDa), sequence length (mer), and chain were obtained from the RCSB PDB database ( https://www.rcsb.org/ ).

#### Absorption, Distribution, Metabolism, Excretion and Toxicity Prediction

Predictions of absorption, distribution, metabolism, excretion, and toxicity of green coffee chemical compounds were performed on Swiss ADME ( http://www.swissadme.ch/ ) and ProTox-II ( https://tox-new.charite.de/protox_II/ ). Physicochemical properties, water solubility, and drug likeness are used to predict the ability of query compounds as a good drug molecule candidate in general, then for the level of toxicity, it is expected to have a low toxicity category consisting of class IV or V. 20 21

#### Virtual Screening

The ability of the query compound activity to bind to the target protein in this study was predicted through molecular docking simulations. Molecular docking can be used to determine the type of activity of a ligand and the pattern of molecular interactions when it binds to the target protein based on the value of binding affinity, the type of binding activity is inhibition or increase according to the research objectives. 22 23 This study used PyRx 0.9.9 version software to identify the binding ability of green coffee compounds to 15 target proteins.

#### Ligand-Protein Interaction

Identification of positions and types of molecular interactions in this study were identified through the Discovery Studio 2016 version of the software. Types of chemical bond interactions such as Van der Waals, hydrogen, hydrophobic, electrostatic, and pi are found in the docked molecular complexes. The interactions formed are weak bonds that play a role in triggering the activity of the target protein. 24

#### Molecular Visualization

The 3D structure resulting from molecular docking is displayed in the form of transparent surfaces, cartoons, sticks, and undergoes coloring selection through PyMol 2.5 version software. The software works with python programming and is used for structural selection or coloring of docked molecular complexes. 25

#### Robusta Green Coffee Bean ( *Coffea Canephora* ) Sample Preparation

RGCB was collected from the Coffee and Cocoa Research Center (Puslitkoka) in Jember, East Java, Indonesia. Fresh RGCB without any indication of damage (physical or disease) were collected by picking and then stored in dark plastic samples and stored in a clean box. The samples obtained were then prepared at the Faculty of Pharmacy Laboratory, Widya Mandala Catholic University. The RGCB are cleaned by washing using running water to remove the attached impurities, then the water bundle is dried using a tissue paper and cut into small pieces (simplicia).

#### Robusta Green Coffee Bean ( *Coffea Canephora* ) Extraction Process

Overall, 350g of RGCB was performed by a single-solvent maceration method using ethanol in a ratio of 1:2 (w/v) with several solvent changes due to solvent saturation. The filtrate and residue were prepared by filtering the maceration products with filter paper. A vacuum rotary evaporator was used to evaporate the filtrate at 4°C. The thick extract obtained from the evaporation results was desalted using the decantation technique which involved mixing the thick extract with ethanol and allowing the salt to settle. Finally, the procedure was repeated until the white tint that indicated the presence of salt in the solvent was no longer visible. The extract was then evaporated once more at 4°C in a vacuum rotary evaporator. Then, for further examination, a 96% ethanol extract of RGCB was produced and stored.

### Phytochemical Analysis of Robusta Green Coffee Bean Ethanol Extract

#### Flavonoid Test

The sample was weighed, then ethanol was used to extract it, cotton was used to filter it, and it was then transferred to another tube. After that, a test with concentrated hydrochloric acid (HCl) was performed by applying the sample's ethanol extract and two drops of concentrated HCl. The extract was shaken vigorously before being mixed with Mg powder and shook again vigorously. When flavonoids were detected using strong HCl reagents, froths formed, and the color of the solution turned orange, the samples were found to be positive. The RGCB ethanol extract was mixed with two drops of 2-normal (2N) sulphuric acid (H _2_ SO _4_ ) and then vigorously shaken. When flavonoids were detected using 2N H _2_ SO _4_ reagents, the color of the solution changed dramatically to yellow, red, or brown, indicating that the samples were positive.

#### Alkaloid Test

After weighing the material, ammoniacal chloroform was used to extract it. Ethanol extract from robusta green coffee beans was applied to tubes A and B. It was then transferred to tubes A and B after being filtered with cotton. Dragendorff's reagents were introduced to tube A, while Wagner's reagents were applied to tube B. If there were reddish deposits, samples in tube A contained positive alkaloids; if there were brownish deposits, samples in tube contained positive alkaloids.

#### Saponin Test

The RGCB ethanol extract was weighed, and ammoniacal chloroform was used to remove it. It was then strained using cotton and transferred to a new tube. The tube was vigorously shaken for 2minutes before adding two drops of 2N HCl. It was well shaken before being allowed to settle for 10minutes to see whether any foam had formed. If there were a lot of froths with a lot of intensity and they were consistent for 10minutes, the sample was certified positive and contained saponins.

#### Triterpenoid and Steroid Test

Weighed RGCB ethanol extract was extracted using ethanol. It was then strained with cloth and heated to dry. The material was then extracted once again using chloroform and water (1:1). Two drops of chloroform were added to the extract on the spotted plate which was then allowed to dry. In addition, the extract was spiked with one drop of concentrated sulfuric acid and one drop of anhydrous acetic acid. Positive triterpenoids samples were confirmed if they had a red or brown discoloration, while positive steroids samples were confirmed if they had a blue, purple, or green discoloration.

#### Antioxidant Activity Test of Robusta Green Coffee Bean Ethanol Extract

For the antioxidant activity test, a DPPH (2,2-difenil-1-pikrilhidrazil) 4×10–4M solution was used which was placed in vials and firmly sealed. To prevent light from entering the vial, it was necessary to cover it with aluminum foil. It then required a 1,000-ppm sample solution which was obtained by dissolving the sample in methanol. The stock and methanol solutions have to be added in the correct volumes for sample testing. DPPH solution of 1mL was added to each test tube which was then left for 30minutes. It was then measured with a ultraviolet-visible (UV-V) spectrometer with a wavelength of 517nm as a last step.

### Antibacterial Activity of Robusta Green Coffee Extract to Peri-implantitis bacteria *Fusobacterium nucleatum* Culture and Preparation

*Fusobacterium nucleatum* (Fn; ATCC22586, United Kingdom) was cultured in Tryptic Soy Broth (TSB) media and incubated for 18 to 24hours at 37°C under anaerobic conditions. Bacterial colonies were taken using a stick which was previously heated with the Bunsen burner then transferred in 3mL of liquid to brain–heart infusion (BHI) media and incubated at 37°C for 18hours. The bacterial suspension was equalized with the McFarland standard of 0.5 (1.5×10 ^9^ ) colony forming unit (CFU)/mL. The suspension is then flattened on the surface of the nutrient agar medium.

### *Aggregatibacter actinomycetemcomitans* Culture and Preparation

Aa (ATCC43718, United Kingdom) cultures was incubated for 24hours at 37°C under anaerobic conditions in BHI media after taken from stock using sterile stick. The cultures were matched to the McFarland standard of 0.5 or the equivalent of 1.5×10 ^8^ CFU/mL. The turbidity of the bacterial suspension by double blind is equated by holding the test tubes next to each other with white background and black stripes. The bacterial suspension is diluted once the turbidity of the bacterial suspension is not matched.

### *P. gingivalis* Culture and Preparation

Pg (ATCC33277, United Kingdom) was cultured in TSB media and incubated for 18 to 24hours at 37°C under anaerobic conditions. Bacterial colonies were taken using a stick which was previously heated over the Bunsen burner then transferred in 3mL of BHI liquid media and incubated at 37°C for 18hours. The bacterial suspension was equalized with the Mc Farland standard of 0.5 (1.5×10 ^9^ CFU/mL). The suspension that has been equalized is taken with a micropipette then flattened on the surface of the nutrient agar media.

### *Prevotella Intermedia* Culture and Preparation

Pi (ATCC25611, United Kingdom) was revived in 5mL thioglycollate broth (BBLTM Fluid, Becton Dickinson and Company) and cultured under anaerobic conditions for 8 days at 35°C (Anaerogen, Oxoid). They were then seeded on blood agar and Wilkins–Chalgren agar and cultured at 35°C for 8 days under anaerobic conditions (Anaerogen, Oxoid). Gram stains were used on isolated colonies to confirm strain shape and purity. Three to five colonies were cultivated in 4mL BHI broth with 5 µg/mL hemin and 1g/mL menadione and incubated for 72hours at 35°C under anaerobic conditions to preenriched microorganisms (Anaerogen, Oxoid). Finally, a 0.5 turbid suspension in the McFarland scale (∼1.5 108CFU/mL) was created from this preenrichment.

### The Peri-implantitis Bacteria Inhibition Zone Analysis

The inhibitory zone was discovered in the Fn, Pg, Aa, and Pi culture plates after administration of RGCB extract with 50, 25, and 12.5% as treatment groups and doxycyline as positive group, respectively, in the paper disk. The inhibition zone was estimated in millimeter units using a digital caliper (Mitutoyo, Japan), and then replicated for each group.

### Minimal Inhibitory Concentration Analysis of Robusta Green Coffea Bean Extract to Peri-implantitis Bacteria

The antibacterial agent stock solution was made by combining 100g of green coffee bean extract with 1mL of thioglycollate (TG) broth medium (100g/1mL). Nine dilutions of the medication were generated using the microdilution method with TG broth medium using conventional protocols for MIC. In addition, 20 L of medication from the stock solution was added to the 380 L of TG broth in the first tube. Also, 200 L of TG broth was added to each of the next nine tubes independently for dilutions. Then 200 L was transferred from the first tube to the first tube holding 200 L of TG broth. This was referred to as a 101% dilution. To create 10 ^2^ dilutions, 200 µL was transferred from the 101 diluted tube to the second tube. For each medication, the serial dilution was performed up to 10 ^9^ times. Then, 10mL of the needed organisms' stock cultures (Fn, Aa, Pg, and Pi) was taken and mixed with 2mL of TG broth. Then 200 µL of the aforesaid culture suspension was added to each serially diluted tube which was then sealed airtight and cultured for 48hours in an anaerobic jar/chamber. The MIC is defined as the lowest concentration of RGCBE ethanol extract in the tube that does not produce turbidity.

### Minimal Bactericidal Concentration analysis of Robusta Green Coffea Bean Extract to Peri-implantitis Bacteria

Following the MIC technique, four dilution tubes with antibacterial sensitivity at lower concentrations were obtained and put into the appropriate culture medium to check microorganism growth. Previously, plates were incubated for 48hours in an anaerobic jar/chamber before colonies were counted.

### Statistical Analysis

After that, all of the research data were summarized and examined descriptively and inferentially. The data are shown in a bar chart with the mean and standard deviation. The data were analyzed using the statistical package for social science (SPSS) version 20.0 for windows which included the normality and homogeneity test ( *p* >0.05), the analysis of variance (ANOVA) difference test, and the post-hoc Tukey's Honest Significant Different (HSD) with a different significance value of *p* <0.05 (IBM corporation, Illinois, Chicago, Illinois, United States).

## Result

**Fig. 1:**
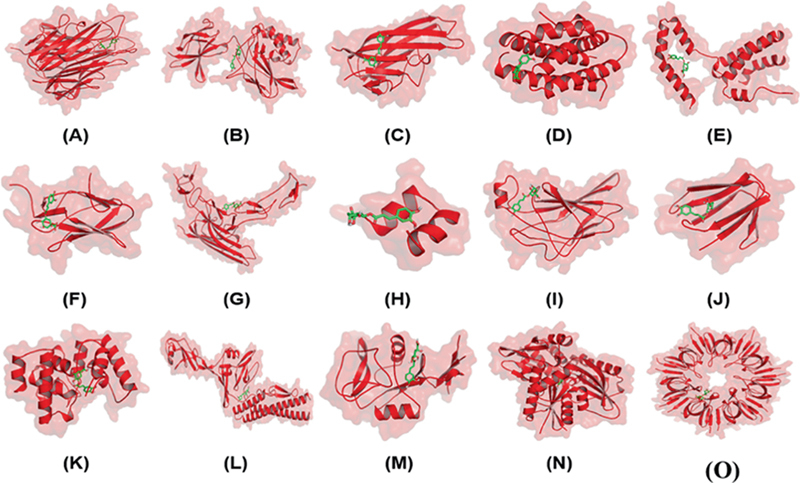
Molecular complex from docking simulation. ( **A** ) TNF-α_Chlorogenic acid, ( **B** ) NF-κB_Chlorogenic acid, ( **C** ) RANKL-RANK_Chlorogenic acid, ( **D** ) IL-6_Chlorogenic acid, ( **E** ) IL-10_Chlorogenic acid, ( **F** ) RUNX2_Chlorogenic acid, ( **G** ) RANKL-OPG_Chlorogenic acid, ( **H** ) Osteocalcin_Chlorogenic acid, ( **I** ) NFATC1_Chlorogenic acid, ( **J** ) TRAP_Chlorogenic acid, ( **K** ) Peptidoglycan_Chlorogenic acid, ( **L** ) Flagellin_Chlorogenic acid, ( **M** ) Dectin_Chlorogenic acid, ( **N** ) Hsp70_Chlorogenic acid, ( **O** ) Hsp10_Chlorogenic acid. IL, interleukin; NF, nuclear factor; TNF, tumor necrosis factor; RANK, receptor activation NF-κB; RANKL, RANK and its ligand.

**Fig. 2:**
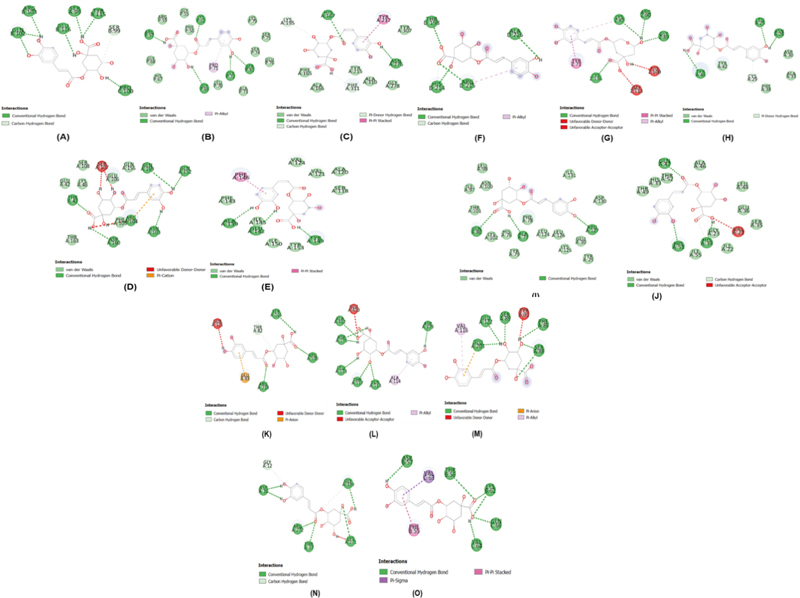
2D visualization of the position and types of chemical bond interactions in the protein-ligand complex. ( **A** ) TNF-α_Chlorogenic acid, ( **B** ) NF-κB_Chlorogenic acid, ( **C** ) RANKL-RANK_Chlorogenic acid, ( **D** ) IL-6_Chlorogenic acid, ( **E** ) IL-10_Chlorogenic acid, ( **F** ) RUNX2_Chlorogenic acid, ( **G** ) RANKL-OPG_Chlorogenic acid, ( **H** ) Osteocalcin_Chlorogenic acid, ( **I** ) NFATC1_Chlorogenic acid, ( **J** ) TRAP_Chlorogenic acid, ( **K** ) Peptidoglycan_Chlorogenic acid, ( **L** ) Flagellin_Chlorogenic acid, ( **M** ) Dectin_Chlorogenic acid, ( **N** ) Hsp70_Chlorogenic acid, ( **O** ) Hsp10_Chlorogenic acid. IL, interleukin; NF, nuclear factor; TNF, tumor necrosis factor; RANK, receptor activation NF-κB; RANKL, RANK and its ligand.

Fifteen target proteins have been obtained from the database ( Table 1 ), based on ADMET predictions that chlorogenic acid and coumaric acid compounds can work as good drug molecules because they meet several drug-likeness parameters, then both compounds can be soluble and allow them to pass through the selectively permeable cell layer if it has a target in the cytoplasmic environment, the toxicity level of the two compounds is also low, namely, class 5 or low toxic ( Table 2 ). The results of molecular docking simulations show that chlorogenic compounds have higher activity than coumaric acid, this is reviewed based on the binding affinity value formed when it binds to all target proteins ( Table 3 ). The docked molecular complexes in this study were displayed using PyMol 2.5 version software with structural selection and staining ( Fig. 1 ). The binding location of the docked protein-ligand complex ( Fig. 2 ) revealed that binding of chlorogenic acid compounds to all target proteins resulted in noncovalent bond interactions consisting of the Van der Waals, pi, and hydrogen bonds ( Table 4 ).

**Table 1:** Protein target from database

No.	Name	Visualization method	Uniprot ID	PDB ID	Resolution (Å)	Weight (kDa)	Sequence length (mer)	Chain
1	TNF-α	X-ray diffraction	P01375	1TNF	2.60	52.11	157	A/B/C
2	NF-κB	Nuclear magnetic resonance (NMR)	P19838	2DBF	–	10.62	100	A
3	RANKL-RANK	X-ray diffraction	A3RF19	3URF	2.70	38.38	162	A
4	IL-6	NMR	P05231	1IL6	–	21.01	185	A
5	IL-10	X-ray diffraction	Q13651	1INR	2.00	18.67	160	A
6	RUNX2	X-ray diffraction	Q13950	6VGE	4.25	62.53	117	D
7	RANKL-OPG	X-ray diffraction	O00300	3URF	2.70	38.38	162	A
8	Osteocalcin	X-ray diffraction	P02818	1Q8H	2.00	5.85	49	A
9	NFATC1	NMR	O95644	1A66	–	27.33	178	A
10	TRAP	X-ray diffraction	Q96HR3	1WAR	2.22	35.48	310	A
11	Peptidoglycan	X-ray diffraction	P46022	2OQO	2.10	23.77	200	A
12	Flagellin	X-ray diffraction	P21184	2ZBI	2.00	60.96	292	A/B
13	Dectin	X-ray diffraction	Q6QLQ4	2CL8	2.80	32.92	139	A/B
14	Hsp70	X-ray diffraction	P0DMW1	1S3X	1.84	42.75	382	A
15	Hsp10	NMR	P38910	6MRD	3.82	27.33	178	C

Abbreviations: NMR, nuclear magnetic resonance; RUNX2, runt related transcription factor; NFATc1, nuclear factor associated t cell-1; TRAP, tartate resistant acid phosphatase; HSP, heat shock protein; IL, interleukin; NF, nuclear factor; TNF, tumor necrosis factor.

**Table 2:** ADMET analysis of chlorogenic acid and coumaric acid

Compounds	Physicochemical properties	Water solubility	Drug likeness	Toxicity
Chlorogenic acid	Formula: C _16_ H _18_ O _9_ Weight: 354.31 g/mol No. Of heavy atoms: 25 No. arom. heavy atoms: 6 Fraction Csp3: 0.38 No. rotatable bonds: 5 No. H-bond acceptors: 9 No. H-bond donors: 6 Molar refractivity: 83.50 TPSA: 164.75 Å ^2^	Log S (ESOL): −1.62 Class: very soluble Log S (Ali): −2.58 Class: soluble Log S (SILICOS-IT): 0.40 Class: soluble	Lipinski: Yes Ghose: No Veber: No Egan: No Muegge: No Bioavailability: 0.11	Predicted LD50: 5,000mg/kg Similarity: 71.21% Predicted toxicity Class: 5 (low toxic)
Coumaric acid	Formula: C _9_ H _8_ O _3_ Weight: 164.16 g/mol No. heavy atoms: 12 No. arom. heavy atoms: 6 Fraction Csp3: 0.00 No. rotatable bonds: 2 No. H-bond acceptors: 3 No. H-bond donors: 2 Molar refractivity: 45.13 TPSA: 57.53 Å ^2^	Log S (ESOL): −2.02 Class: soluble Log S (Ali): −2.27 Class: soluble Log S (SILICOS-IT): −1.28 Class: soluble	Lipinski: yes Ghose: yes Veber: yes Egan: yes Muegge: no Bioavailability: 0.85	Predicted LD50: 2,850mg/kg Similarity: 100% Predicted toxicity Class: 5 (low toxic)

Abbreviations: ADMET, absorption, distribution, metabolism, excretion, and toxicity; TPSA, topological polar surface area.

**Table 3:** The molecular docking result to molecule target

Protein	Autogrid						Binding affinity (kcal/mol)	
	Center (Å)			Dimensions (Å)			Chlorogenic acid	Coumaric acid
	X	Y	Z	X	Y	Z		
TNF-α	19.968	49.675	39.930	80.739	58.243	58.256	–9.6	–5.8
NF-κB	42.464	14.683	38.036	90.709	67.390	51.935	–6.5	–5.5
RANKL-RANK	8.830	–0.536	17.364	47.953	57.932	51.740	–6.5	–5.3
IL-6	2.675	–20.084	8.908	58.092	62.897	43.139	–6.8	–5.4
IL-10	13.263	21.832	5.096	58.882	38.528	85.481	–5.8	–5.0
RUNX2	–50.956	39.759	–15.612	64.939	22.064	47.175	–6.3	–4.5
RANKL-OPG	–2.998	–3.671	23.788	95.207	98.636	77.806	–6.8	–5.5
Osteocalcin	8.075	25.301	22.863	31.622	16.932	12.741	–4.8	–4.4
NFATC1	15.501	–7.918	1.696	68.461	53.410	57.713	–8.2	–6.3
TRAP	68.142	–24.322	17.856	33.318	38.191	40.670	–6.2	–5.0
Peptidoglycan	37.648	37.735	21.932	64.278	40.527	45.926	–7.0	–5.7
Flagellin	–23.829	37.749	33.866	149.906	40.586	98.210	–7.5	–5.8
Dectin	43.337	20.890	45.579	55.338	39.093	31.835	–7.8	–5.0
Hsp70	17.349	28.677	16.059	81.800	54.749	57.266	–9.2	–6.6
Hsp10	–40.185	60.186	24.034	107.037	65.608	86.197	–7.9	–5.7

Abbreviations: IL, interleukin; NF, nuclear factor; TNF, tumor necrosis factor; RANK, receptor activation NF-κB; RANKL, RANK and its ligand; RUNX2, runt related transcription factor; NFATc1, nuclear factor associated t cell-1; TRAP, tartate resistant acid phosphatase; HSP, heat shock protein.

**Table 4:** Results of molecular interaction analysis

Ligan protein	Chemical interaction
Chlorogenic acid_TNF-α	Hydrogen: Gln102, Arg103, Glu116, Ser99, Tyr115, Ser99, Pro100
Chlorogenic acid_NF-κB	Hydrogen: Arg57, Lys52, Ser81, Gly72, Gly69 Van der Waals: Arg59, Gly55, Phe56, Lys79, Ser75, Ser74, Glu76, Ala73, Leu70, Gly68, His67 Pi: Pro71
Chlorogenic acid_RANKL-RANK	Hydrogen: His167, Asn276, Phe311, Lys195 Van der Waals: Phe165, Ala166, Ala310, Tyr215, Tyr307, Gly278 Pi: Tyr217
Chlorogenic acid_IL-6	Hydrogen: Gln159, Gln152, Thr43, Arg104, Asn103, Thr43, Asp160 Van der Waals: Glu42, Thr163, Lys46, Ser108, Glu106, Glun156 Pi: Phe105, Arg104 Unfavorable: Se107
Chlorogenic acid_IL-10	Hydrogen: Ala139, Glu142, Tyr149 Van der Waals: Val124, Val121, Ala120, Ser118, Phe143, Ile145, Ile150, Tyr153 Pi: Phe146
Chlorogenic acid_RUNX2	Hydrogen: Thr198, His214, Arg215, Ala216 Pi: Arg215
Chlorogenic acid_RANKL-OPG	Hydrogen: Lys87, Arg90, Gly89 Pi: Tyr71, Lys87 Unfavorable: Gly113, His100
Chlorogenic acid_Osteocalcin	Hydrogen: Tyr46, Leu25, Asn26, Van der Waals: Tyr42, Ala33, Phe38, Asp30 Pi: Cys29
Chlorogenic acid_NFATC1	Hydrogen: Gln80, Ala77, Arg127 Van der Waals: Leu98, Ile97, Asn100, Thr101, Lys102, His76, Tyr79, Phe78, Leu124, Leu126, Lys125, Glu30, Tyr29, Asp130, Ile131
Chlorogenic acid_TRAP	Hydrogen: Gln47, His51, His34 Van der Waals: Thr49, His33, Thr52, Leu44, Glu36, Ser35, Gly23, Ile22, Ile55 Unfavorable: Val21
Chlorogenic acid_Peptidoglycan	Hydrogen: Ser116, Arg100, Thr82, Arg218 Pi: Glu83 Unfavorable: Gln121
Chlorogenic acid_Flagellin	Hydrogen: Ala412, Asp171, Leu173, Thr117, Gln113, Asp379 Pi: Ala114 Unfavorable: Glu410
Chlorogenic acid_Dectin	Hydrogen: Glu203, Glu122, Ser89, Phe86, Ser88 Pi: Glu203, Val116 Unfavorable: Tyr91
Chlorogenic acid_Hsp70	Hydrogen: Gly12, Asp10, Gly339, Arg272, Thr37, Lys271
Chlorogenic acid_Hsp10	Hydrogen: Asp57, Thr56, Lys62, Gln66, Val64 Pi: Val63, Phe55

Abbreviations: IL, interleukin; NF, nuclear factor; TNF, tumor necrosis factor; RANK, receptor activation NF-κB; RANKL, RANK and its ligand; RUNX2, runt related transcription factor; NFATc1, nuclear factor associated t cell-1; TRAP, tartate resistant acid phosphatase; HSP, heat shock protein.

**Fig. 3:**
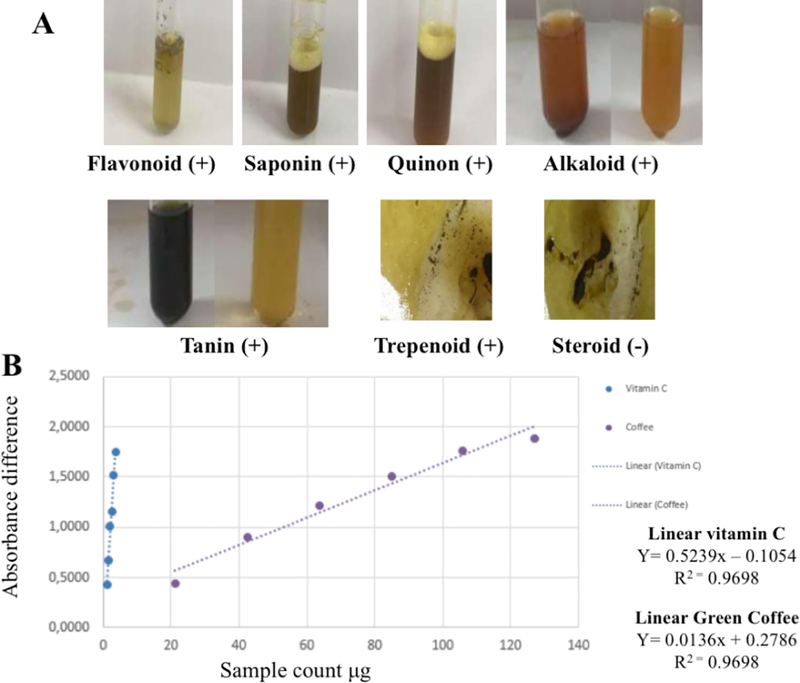
( **A** ) The phytochemical analysis of RGCB ethanol extract contained flavonoid, saponin, quinon, alkaloid, tannin, trepenoid but not steroid. ( **B** ) The antioxidant test of RGCB ethanol extract has high antioxidant ability than vitamin C. RGCB, robusta green coffee bean.

**Fig. 4:**
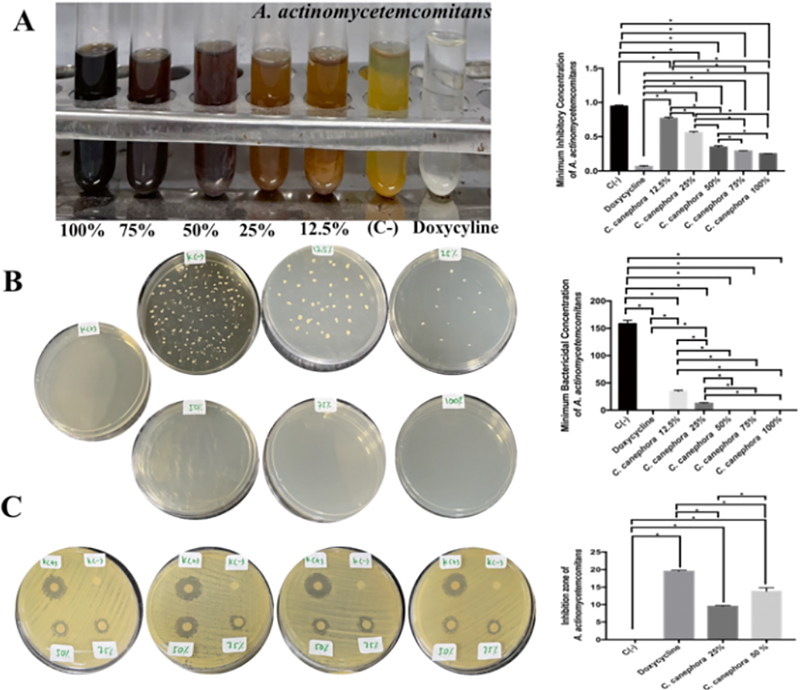
The antibacterial activity of RGCB ethanol extract toward *Aggregatibacter actinomycetemcomitans* . ( **A** ) The MIC of *A. actinomycetemcomitans* after administration of RGCB ethanol extract showed 50, 75, and 100% has strong antibacterial activity. ( **B** ) The MBC of *A. actinomycetemcomitans* after administration of RGCB ethanol extract showed 50, 75, and 100% has strong antibacterial activity. ( **C** ) The inhibitory zone using disk diffusion examination on *A. actinomycetemcomitans* after administration of RGCB ethanol extract showed 50% better than 25% antibacterial activity. *Information: significant different at *p* <0.05. MBC, minimum bactericidal concentration; MIC, minimum inhibitory concentration; RGCB, robusta green coffee bean.

The phytochemical analysis of RGCB ethanol extract contained flavonoid, saponin, quinone, alkaloid, tannin, and trepenoid but not steroid ( Fig. 3A ). The antioxidant test of RGCB ethanol extract has high antioxidant ability as much as 59.69 µg than vitamin C as much as EC _50_ :2.28 µg ( Fig. 3B ). In this study, RGCB ethanol extract has MIC and MBC and can inhibit the growth of Aa, Pg, Fg, and Pi with the bacteria which involve during peri-implantitis course. The most MIC and MBC of Aa was found in doxycycline treatment followed by RGCB ethanol extract of 100, 75, and 50% with significant different ( *p* =0.0001 and<0.05; Fig. 4A and B ). The most extensive zone of inhibition of Aa was found in doxycycline treatment followed by RGCB ethanol extract of 100, 75, and 50%. There was a significant difference between the treatment groups on the inhibition zone of Aa *.* There was significant difference between 50 and 25% of RGCB ethanol extractions inhibiting Aa growth ( *p* =0.0001 and<0.05; Fig. 4C ).

**Fig. 5:**
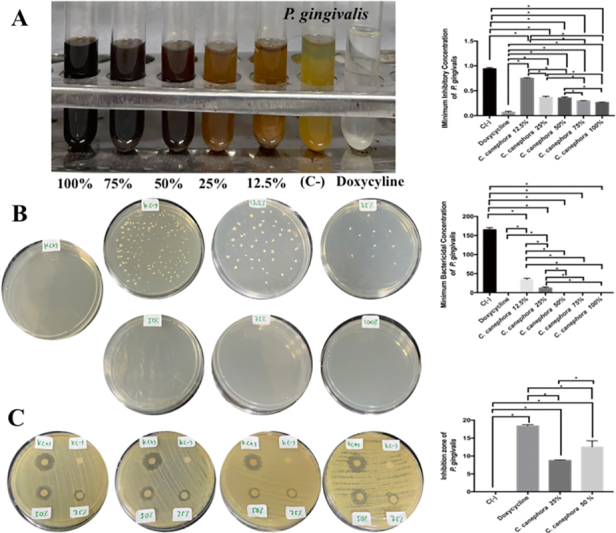
The antibacterial activity of RGCB ethanol extract toward *Porphyromonas gingivalis* . ( **A** ) The MIC of *P. gingivalis* after administration of RGCB ethanol extract showed 50, 75, and 100% has strong antibacterial activity. ( **B** ) The MBC of *P. gingivalis* after administration of RGCB ethanol extract showed 50, 75, and 100% has strong antibacterial activity. ( **C** ) the inhibitory zone using disk diffusion examination on *P. gingivalis* after administration of RGCB ethanol extract showed 50% better than 25% antibacterial activity. *Information: significant different at *p* <0.05. MBC, minimum bactericidal concentration; MIC, minimum inhibitory concentration; RGCB, robusta green coffee bean.

**Fig. 6:**
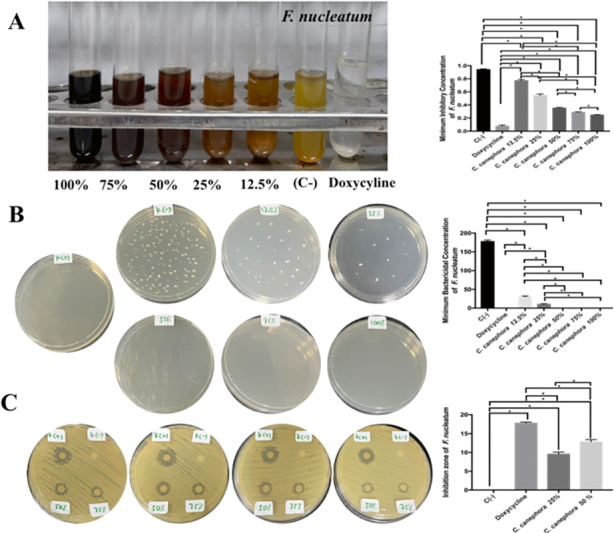
The antibacterial activity of RGCB ethanol extract toward *Fusobacterium nucleatum* . ( **A** ) the MIC of *F. nucleatum* after administration of RGCB ethanol extract showed 50%, 75%, 100% has strong antibacterial activity. ( **B** ) the MBC of *F. nucleatum* after administration of RGCB ethanol extract showed 50, 75, and 100% has strong antibacterial activity. ( **C** ) the inhibitory zone using disk diffusion examination on *F nucleatum* after administration of RGCB ethanol extract showed 50% better than 25% antibacterial activity. *Information: significant different at *p* <0.05. MBC, minimum bactericidal concentration; MIC, minimum inhibitory concentration; RGCB, robusta green coffee bean.

**Fig. 7:**
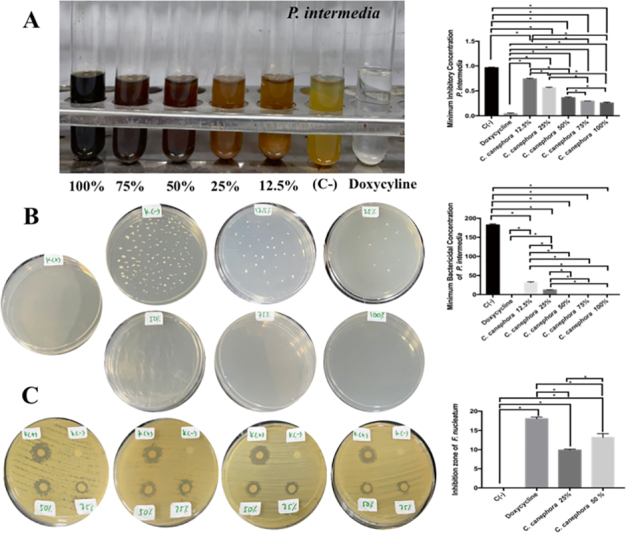
The antibacterial activity of RGCB ethanol extract toward *Prevotella intermedia* . ( **A** ) the MIC of *P. intermedia* after administration of RGCB ethanol extract showed 50, 75, and 100% has strong antibacterial activity. ( **B** ) the MBC of *P. intermedia* after administration of RGCB ethanol extract showed 50, 75, and 100% has strong antibacterial activity. ( **C** ) the inhibitory zone using disk diffusion examination on *P. intermedia* after administration of RGCB ethanol extract showed 50% better than 25% antibacterial activity. *Information: significant different at *p* <0.05. MBC, minimum bactericidal concentration; MIC, minimum inhibitory concentration; RGCB, robusta green coffee bean.

The most MIC and MBC of Pg was found in doxycycline treatment followed by RGCB ethanol extract of 100, 75, and 50% with significant different ( *p* =0.0001 and<0.05; Fig. 5A and B ). The most extensive zone of inhibition of Pg was found in doxycycline treatment followed by RGCB ethanol extract of 100, 75, and 50%. There was a significant difference between the treatment groups on the inhibition zone of Pg *.* There was significant different between 50 and 25 of RGCB ethanol extractions inhibiting Pg ( *p* =0.0001 and<0.05; Fig. 5C ). The most MIC and MBC of Fn was found in doxycycline treatment followed by RGCB ethanol extract of 100, 75, and 50% with significant different ( *p* =0.0001 and<0.05; Fig. 6A and B ). The most extensive zone of inhibition of Fn was found in doxycycline treatment followed by RGCB ethanol extract of 100, 75, and 50%. There was a significant difference between the treatment groups on the inhibition zone of Fn. There was significant difference between 50 and 25% of RGCB ethanol extract inhibiting Fn ( *p* =0.0001 and<0.05; Fig. 6C ). The most MIC and MBC of Pi was found in doxycycline treatment followed by RGCB ethanol extract of 100, 75, and 50% with significant difference ( *p* =0.0001 and<0.05; Fig. 7A and 7B ). The most extensive zone of inhibition of Pi was found in doxycycline treatment followed by RGCB ethanol extract of 100, 75, and 50%. There was a significant difference between the treatment groups on the inhibition zone of Pi. There was significant different between 50 and 25% of RGCB ethanol extract inhibiting Pi ( *p* =0.0001 and<0.05; Fig. 7C ).

## Discussion

Molecular docking simulations are used to predict the mechanism of binding of chlorogenic acid and coumaric acid to proteins. The simulation seeks to create negative energy by determining the level of binding ability of a ligand to a protein domain based on the binding affinity value of the ligand-protein stable complex. 26 When a protein interacts with a ligand, binding affinity is established. This energy is created by a reversible reaction at constant temperature and pressure, according to thermodynamic rules. 27 The grid in the docking simulation aids in the direction of ligand binding to the target protein. 28

Peri-implantitis is an inflammatory condition that affects the periodontal tissue around a dental implant that has been osseointegrated. 29 In the peri-implant crevicular fluid of implants with peri-implantitis, there were higher amounts of proinflammatory cytokines than in healthy implants. When periimplantitis was compared with healthy peri-implant mucosa, the secretion of IL-1b, IL-6, and TNF-α was significantly higher. 10 12 TNF-α promotes bone resorption either directly by encouraging osteoclast differentiation and maturation and increasing their resorptive activity or indirectly by interacting with the RANKL-RANK. 8 The activity of chlorogenic acid compounds in green coffee allows it to be anti-inflammatory through inhibition of regulation or decrease in the activity of proinflammatory proteins, such as TNF-α, NF-κB, RANKL-RANK, and IL-6, which can then trigger upregulation of anti-inflammatory proteins, such as IL-10, then activity chlorogenic acid can increase osteoblast and osteoclast activity through RUNX2, RANKL-OPG, osteocalcin, NFATC1, and TRAP, antibacterial potential is also found in chlorogenic acid through inhibition of peptidoglycan, flagellin, and dectin activity, and chlorogenic acid can also be an antioxidant through upregulation of Hsp70 and Hsp10. Hydrogen bonds, hydrophobicity, the Van der Waals, and pi, all play a role in the docking complex's weak bond interactions which help to initiate the creation of specific biological activities. 30 Overall, weak binding interactions can help build stable ligand-protein complexes and trigger activity responses on target proteins including enhancement and inhibition.

Lipopolysaccharide (LPS) is a prominent component of gram-negative bacteria's outer membrane, including Pi. It can cause a variety of proinflammatory cytokines to be produced and released by a variety of host cells. 31 Aa, an opportunistic periodontopathogen with various virulence factors, is able to resist the clearance attempts because of its protective extracellular matrix and due to the presence of resistant cells. Fimbriae are important in the initial adhesion of Aa to dental surfaces and in biofilm formation. 32 If peri-implantitis is not discovered and treated early, bone deterioration can extend the entire length of the implant, causing it to lose stability. 10 Phytochemicals constituent of green coffee involve phenolic compounds and their derivatives (such as chlorogenic acid), alkaloids (especially caffeine), diterpenoid alcohols (such as cafestol and kahweol), carbohydrates, lipids, and volatile and heterocyclic. 33 The alkaloid ingredient in RGCB ethanol extract may prevent peri-implantitis by breaking peptidoglycan in the bacterium's cell wall. Green coffee flavonoids damage bacterial cell membrane integrity by preventing the formation of bacterial extracellular protein complexes. Chlorogenic acid, the most abundant polyphenol in green coffee, has been linked to protein denaturation and cell membrane, microsome, and lysosome damage. In bacteria, cholinergic acid can inhibit enzymes and interfere with coaggregation. 34 Based on *in silico,* cholinergic acid also have interfered another component of bacteria, namely, peptidoglycan, flagellin, and dectin, to inhibit bacterial activity.

Coffee beans contain a lot of phenolic compounds, especially chlorogenic acid that plays a role as an antioxidant. 35 36 The antioxidant activity of coffee beans depends on the ability to scavenge reactive oxygen species (ROS). 36 Through the production of reactive oxygen species, oxidative stress can boost the expression of Hsp10 and Hsp70 (ROS). Hsp10 and Hsp70 levels must be raised in order for cells to recover and maintain equilibrium. Hsp molecules act as chaperones, assisting in folding, intracellular protein trafficking, denatured protein handling, and cell repair. 37

Polyphenol contained in green coffee is reported may be an antioxidant agent and scavenge free radical. 38 Meanwhile, the roasting process may reduce the antioxidant capacity of coffee beans by destroying numerous of their phenolic components. Green coffee antioxidant activity may also decrease inflammation by reducing free radicals. Inflammation inhibition may diminish osteoclast activation and promote osteogenesis. 39 RGCB consists of chlorogenic acid (CGA) and caffeic acid which are believed to act as an antioxidant and take part in wound healing process. CGA stimulates the mobilization of macrophages, may indirectly increase the ability of macrophage phagocytosis because it affects the secretion of interferon (IFN)-γ that act as macrophage activators. These cytokines may activate macrophages, so that macrophages release other cytokines to activate lymphocytes and causes inflammation where there is a focus of both these cells stimulate each other to destroy the antigen. 40 Robusta coffee bean extract contains active compounds, namely, polyphenols, alkaloids, and saponins which act as an anti-inflammatory agent by inhibiting Polymorphonuclear leukocytes (PMN)s and macrophage for producing matrix metalloproteinase (MMP), prostaglandine E2 (PGE2), and pro-inflammatory cytokines such as IL-1, IL-6, and TNF-α. The administration of robusta coffee bean polyphenol extract was effective in reducing the degree of inflammation which was characterized by a decrease in TNF-α expression. Decreased production of proinflammatory cytokines leads to inhibition of osteoclast formation. 41 42 43 RUNX2 expression in osteoblasts can be increased by chlorogenic acid in green coffee due to antioxidant action which is significant in promoting osteoblastic activity through particular receptors. 42 43 In the early phases of osteoblast differentiation, increased RUNX2 expression can stimulate the induction of bone matrix gene expression, such as osteocalcin (OCN), alkaline phosphatase (ALP), and others. 44 45 Chlorogenic acid prevents RANKL-induced osteoclastogenesis associated with downregulation of nuclear factor kappa beta (NFKB), Jun-amino-terminal kinase (JNK), extracellular signal-regulated kinase (ERK), p38 mitogen-activated protein kinases (P38 MAPK) activation, and suppression of c-Fos and NFATc1 expression, leading to decreased expression of Osteoclast-associated receptor (OSCAR) and Tartate Resistant Acid Phosphatase (TRAP) 46 RGCB ( *C. canephora* ) may promising phytotherapy that possessed antioxidant, anti-inflammatory, antibone resorption, proosteogenic, and antibacterial ability. In addition, RGCB ethanol extract can be a potential therapeutic candidate to prevent bone resorption and bone rejection in mini-implants. Therefore, this study result is limited to *in silico* study and *in vitro* study using American Type Culture Collection (ATCC) bacteria related to peri-implantitis, further study is still needed to investigate the exact mechanism regarding the bone regeneration of peri-implantitis after administration of RGCB ethanol extract *in vivo* with various experimental methods.

## Conclusion

Based on the findings of this investigation, it can be inferred that RGCB ethanol extract has a high antioxidant capacity and that 50% RGCB ethanol extract may be effective against periimplantitis bacteria *in vitro.* In addition, an *in-silico* analysis found that chlorogenic acid in RGCB has antioxidant, antibacterial, anti-inflammatory, antibone resorption, and proosteogenic properties. Further research on the potential of chlorogenic acid in RGCB in a peri-implantitis model *in vivo* is urgently needed.
